# Preoperative staging of cervical cancer: time to shift from cystoscopy to MRI

**DOI:** 10.1007/s00330-025-12039-5

**Published:** 2025-10-04

**Authors:** Matteo Bonatti, Riccardo Valletta, Luca D’Erme, Miriam Dolciami, Roberta Chianura, Pietro Paolo Maria Azzaro, Chiara Innocenzi, Nicolò Bizzarri, Vincenzo Vingiani, Giovanni Negri, Martin Steinkasserer, Sara Notaro, Francesca Vanzo, Elena Magri, Benedetta Gui, Evis Sala, Giacomo Avesani

**Affiliations:** 1 Department of Radiology, Hospital of Bolzano (SABES-ASDAA), Teaching Hospital of Paracelsus Medical University (PMU), 5 Böhler Street, 39100 Bolzano, Italy; 2 Department of Imaging and Radiation Oncology, Fondazione Policlinico Universitario A. Gemelli IRCCS, Largo Agostino Gemelli 8, 00168 Roma, Italy; 3 Catholic University of the Sacred Heart, Largo Francesco Vito 1, 00168 Rome, Italy; 4 Department of Women and Child Health and Public Health, Fondazione Policlinico Universitario A. Gemelli-IRCCS, Largo Agostino Gemelli 8, 00168 Roma, Italy; 5 Department of Pathology, Hospital of Bolzano (SABES-ASDAA), Teaching Hospital of Paracelsus Medical University (PMU), 5 Böhler Street, 39100 Bolzano, Italy; 6 Department of Gynecology and Obstetrics, Hospital of Bolzano (SABES-ASDAA), Teaching Hospital of Paracelsus Medical University (PMU), 5 Böhler Street, 39100 Bolzano, Italy; 7 Department of Radiation Oncology, APSS Trento, L.go Medaglie d’Oro 9, 38122 Trento, Italy

**Keywords:** Magnetic resonance imaging, Uterine cervical neoplasms, Neoplasm staging, Urinary bladder, Survival

## Abstract

**Objectives:**

To evaluate the impact of MRI-defined bladder wall invasion from uterine cervical cancer (CC) on disease recurrence and overall survival.

**Materials and methods:**

IRB-approved multicenter retrospective study including women who underwent staging MRI for histologically confirmed CC (Jan 2015–Dec 2020). Image analysis was independently performed by two radiologists. Bladder wall invasion was diagnosed if ≥ 3 of the following criteria were met: loss of the cervix-bladder fat plane, bladder wall thickening, loss of bladder wall T2-hypointensity, and presence of endoluminal tumor growth. MRI findings were compared with endoscopy/cytology. The impact of MRI-defined bladder wall invasion on tumor recurrence and survival was assessed using logistic regression. Survival curves were compared using the log-rank test.

**Results:**

We included 214 women with a median age of 55 (IQR 47–65) years. MRI-defined bladder wall invasion was observed in 21.5% of patients. Cystoscopy revealed bladder mucosal infiltration in 7.0% of patients, all of whom demonstrated MRI-defined bladder wall invasion. No patients without MRI-defined bladder wall invasion showed mucosal infiltration on cystoscopy/cytology. The median follow-up was 32 months: 46.7% of patients had recurrence, and 23.4% had CC-related death. On logistic regression, MRI-defined bladder wall invasion was an independent risk factor for tumor recurrence (OR 2.24, *p* = 0.047) and mortality (OR 3.55, *p* = 0.006), whereas cystoscopy-defined bladder mucosa infiltration was not. The log-rank test demonstrated a significant difference in survival between patients with and without MRI-defined bladder wall invasion (*χ*² = 15.40, *p* = 0.0001).

**Conclusions:**

MRI-defined bladder wall invasion represents an independent negative prognostic factor in patients with cervical cancer.

**Key Points:**

***Question*** *The prognostic significance of MRI-defined bladder wall invasion in patients with cervical cancer remains unclear with respect to disease recurrence and survival*.

***Findings*** *Bladder wall invasion identified on MRI is an independent predictor of tumor recurrence and tumor-specific mortality, whereas mucosal infiltration detected via cystoscopy is not*.

***Clinical relevance*** *MRI can safely replace cystoscopy in the preoperative staging of patients with uterine cervical cancer. This approach can reduce costs and expedite the staging process*.

**Graphical Abstract:**

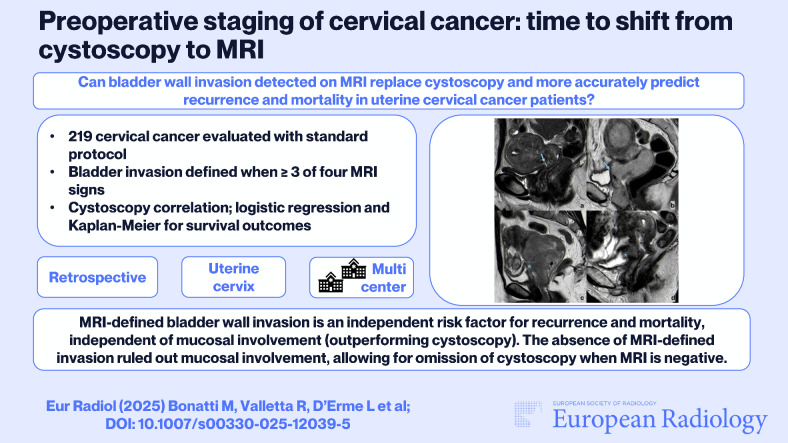

## Introduction

Uterine cervical cancer (CC) is staged according to the International Federation of Gynecology and Obstetrics (FIGO) staging system, which was revised in 2018 to incorporate cross-sectional imaging into the preoperative work-up [1–3]. In developed countries, pelvic MRI is performed for local staging in patients clinically suspected of having stage IB or higher neoplasms, whereas CT or PET/CT is used to exclude distant metastases [4–9]. Accurate preoperative staging is crucial for both treatment planning (e.g., stage IA, IB1, and IIA1 lesions are eligible for surgical treatment, while more advanced cases are candidates for platinum-based chemotherapy with concurrent radiotherapy) and prognosis [10–14].

Despite the updates in 2018, clinical pelvic examination, including colposcopy and cystoscopy, should remain part of the preoperative work-up for staging of CC [15–18]. Indeed, according to the 2018 FIGO guideline, stage IVA is confirmed only through biopsy-proven bladder or rectal mucosal infiltration, leaving MRI without a definitive role in this assessment. Some studies have already demonstrated that MRI has an excellent negative predictive value for ruling out bladder/rectal mucosal infiltration, but its positive predictive value is less reliable, ranging from 17% to 69% when compared to endoscopy [18–24]. A recently published meta-analysis concluded that endoscopy may be avoided in patients with no MRI evidence of bladder invasion, but that it should still be performed to confirm mucosal involvement in patients with MRI signs of invasion [19]. Consequently, some centers stopped routinely performing cystoscopy in patients with no suspicion of bladder invasion at MRI. On the other hand, since bladder invasion in CC occurs “ab extrinseco”, the absence of mucosal infiltration on endoscopic biopsy does not exclude bladder wall involvement.

The aim of our study was to evaluate the prognostic value of MRI-defined bladder wall invasion on disease-free survival and overall survival in patients with uterine cervical cancer and to compare this with the prognostic value of endoscopy-detected bladder mucosal infiltration.

## Materials and methods

### Patients’ population

This retrospective, multicenter cohort study was approved by the Ethical Committee for Clinical Experimentation of the Autonomous Province of Bolzano (protocol 64-2024). Following the committee’s determination, written informed consent was sought a posteriori from living patients who could be contacted. For patients who were deceased or could not be reached despite reasonable attempts, the requirement for informed consent was waived by the committee because of the retrospective nature of the study. All data were anonymized before analysis and handled in accordance with the Declaration of Helsinki and applicable data-protection regulations.

**Fig. 1:**
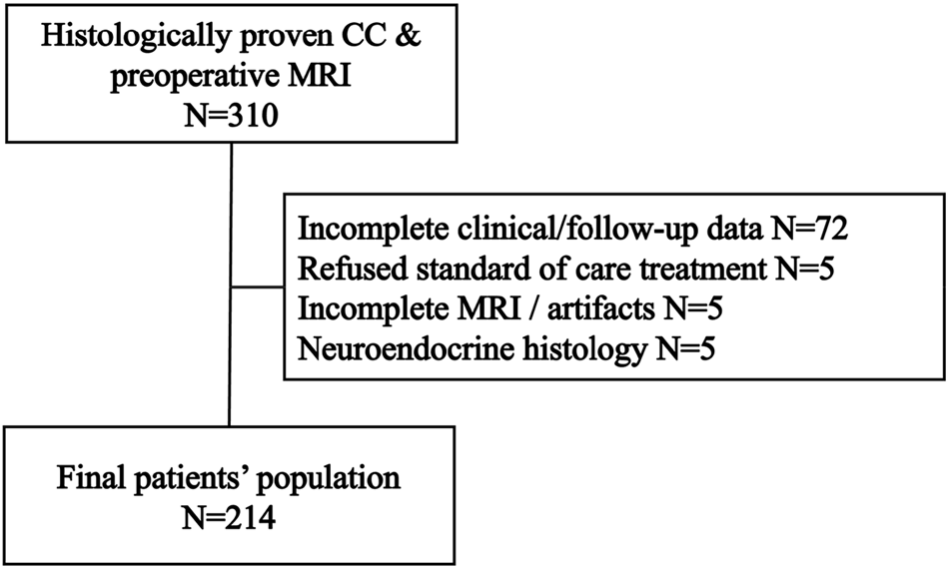
STARD flowchart showing patients’ population building. (CC, uterine cervical cancer)

301 patients who underwent pelvic MRI for local staging of locally advanced histologically confirmed uterine cervical cancer at Bolzano Central Hospital (Center A) or IRCCS Policlinico Universitario Agostino Gemelli, Rome (Center B) between January 2015 and December 2020 were considered for inclusion in the study (Fig. 1). Inclusion criteria were the availability of clinical and radiological follow-up data, as well as surgical, radiotherapeutic, or oncological treatment performed according to the standard of care. Exclusion criteria included incomplete MRI examinations or insufficient image quality due to motion artifacts and neuroendocrine histology.

### Imaging protocol

MR examinations were performed on 1.5-T scanners (Ingenia, Philips Healthcare, in Center A, and Signa, GE, in Center B) following the recommendations of the European Society of Urogenital Radiology (ESUR) [25]. Patients were instructed to void 1 h before the examination. To minimize peristaltic motion artifacts, 20 mg of Hyoscine Butylbromide (Buscopan) was administered intramuscularly, unless contraindicated, immediately before entering the magnet room. Each examination included high-resolution Fast Spin Echo (FSE) T2-weighted images acquired on three orthogonal planes according to the cervical longitudinal axis, high b-value axial diffusion-weighted images of the pelvis, and large field-of-view (FoV) FSE T1-weighted or fat-saturated FSE T2-weighted images of the pelvis and abdomen up to the renal hila.

### Image analysis

**Fig. 2:**
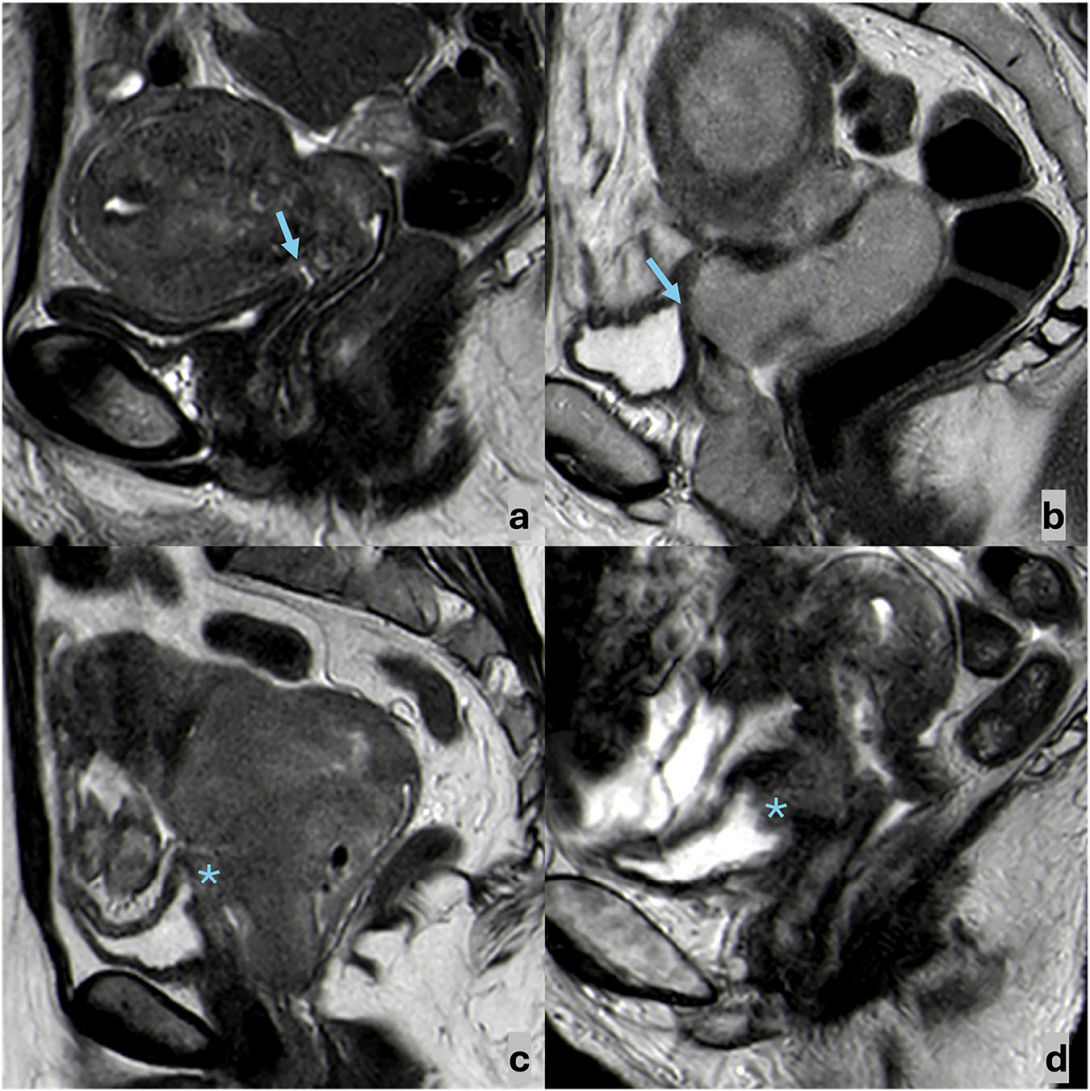
Sagittal T2-weighted FSE images showing the possible appearances of bladder wall–cervical cancer relation. **a** A fatty cleavage plane (arrow) may be observed between CC and the bladder, which shows a thin T2-hypointense wall. **b** The fatty cleavage plane between CC and the bladder is obliterated (arrow), but the bladder wall appears thin and T2-hypointense. **c** The fatty cleavage plane between CC and the bladder is obliterated, and the bladder wall appears thickened, with intermediate signal intensity (star). **d** The fatty cleavage plane between CC and the bladder is obliterated, the bladder wall appears thickened, with intermediate signal intensity, and neoplastic tissue protrudes in the bladder lumen (star)

Image analysis was performed by two radiologists (MB and GA), with 14 and 9 years of experience in pelvic MRI, who were aware of the histological diagnosis of cervical cancer (CC) but blinded to other clinical information, on a workstation using commercially available PACS (Syngo.plaza, Siemens, and Carestream, Philips). The two readers independently evaluated four MRI parameters for assessing CC-bladder wall relationship: (1) loss of the fatty cleavage plane between the neoplasm and the bladder (yes/no), (2) presence of bladder wall thickening (yes/no), (3) loss of the physiological T2-hypointensity of the bladder wall (yes/no), and (4) presence of endoluminal tumor growth (yes/no) (Fig. 2); discrepancies were then solved by consensus. Given the fact that bladder invasion by CC happens ab extrinsic, that bladder wall thickening can also occur in the absence of neoplastic infiltration, and that a focal signal alteration in an otherwise normal bladder wall does not indicate tumor involvement, bladder wall infiltration was considered present if ≥ 3 of the above findings were observed. Moreover, the two readers evaluated in consensus lesions’ maximum diameter (the largest diameter measurable on any of the multiplanar T2-weighted images), as well as the presence of upper 2/3 vaginal invasion (yes/no), lower 1/3 vaginal invasion (yes/no), parametrial involvement (yes/no), pelvic wall involvement (yes/no), hydronephrosis (yes/no), pelvic lymphadenopathies (yes/no), para-aortic lymphadenopathies (yes/no), rectal invasion (yes/no), distant metastases (yes/no). Lymphadenopathy was defined as lymph nodes with a shortest diameter ≥ 8 mm (in the pelvis) or ≥ 10 mm (retroperitoneal). The MRI FIGO stage was subsequently assigned.

Two gynecologists retrieved all relevant clinical data from the Institutional databases, including clinical staging, cystoscopy/urinary cytology reports, treatment type, post-treatment complications, tumor recurrence, and survival data. MRI findings were compared with endoscopic/cytologic findings, and both were analyzed in relation to post-treatment complications, tumor recurrence, and survival outcomes.

### Statistical analysis

Considering the average survival rates reported in the literature [11], a statistical power of 80%, a two-tailed alpha error corrected for Bonferroni at 0.05, an asymmetric allocation ratio of 3:1 (stage IVa cases characterized by bladder infiltration, are less common than stages IIb-IIIc), and assuming no dropouts due to the retrospective nature of the study, the calculated sample size was 200 patients.

Continuous variables are expressed as mean ± standard deviation (SD) or medians with interquartile ranges (IQR), according to distribution normality. Categorical variables are presented as proportions and percentages. MedCalc (version 20, MedCalc Software) was used for all statistical analyses. Normality was assessed using the D’Agostino-Pearson test.

Interobserver agreement in CC-bladder wall relationship evaluation was calculated by means of the kappa statistic.

Fourteen patients with MRI-stage I neoplasms were excluded from further analyses, and 7 patients who died from causes unrelated to cancer were excluded from the mortality analysis. Both univariate and multivariate analyses were performed to assess the influence of different variables on the probability of recurrence and mortality. An independent-samples two-tailed *t*-test or the Mann–Whitney *U*-test was applied, as appropriate, to compare continuous variables, while Fisher’s exact test and the chi-square test were used for dichotomous variables. Variables with *p*-values < 0.10 at univariate analysis were considered for inclusion in the multivariate logistic regression analysis; however, we decided to exclude the tumor’s largest diameter and stage, as they were significantly correlated with almost every other considered variable (age, tumor stage, distant metastases, lymphadenopathies, and surgical treatment). The model’s calibration was assessed using the Hosmer-Lemeshow test.

Kaplan–Meier curves and the log-rank test were used to compare survival rates between different subgroups of patients.

A *p*-value < 0.05 was considered statistically significant.

## Results

### Descriptive analysis

The study population included 214 women with a median age of 55 (IQR 47–65) years: 103/214 (48.1%) from in Center A, and 111/214 (51.9%) from Center B.

At histology, 185/214 (86.4%) of the neoplasms were classified as squamous cell carcinoma and 29/214 (13.6%) as adenocarcinoma (Table 1). Tumor grade was G1 in 12/214 (5.6%) cases, G2 in 110/214 (51.4%), and G3 in 92/214 (43.0%).

**Table 1:** Patients’ population

Parameter		Data
Number of patients		214
Median age (years)		55 (IQR 47–65)
Histological type	Squamocellular	185 (86.4%)
	Adenocarcinoma	29 (13.6%)
MRI-FIGO stage	I	14 (6.5%)
	II	60 (28.0%)
	III	91 (42.5%)
	IV	49 (22.9%)

On MRI, the included lesions had a median maximum diameter of 52 mm (IQR 38–62). The upper two-thirds of the vagina were infiltrated in 183/214 (85.5%) of cases, the parametria in 177/214 (82.7%), the lower third of the vagina in 21/214 (9.8%), the pelvic walls in 8/214 (3.7%), and the rectal wall in 11/214 (5.1%). Hydronephrosis was observed in 27/214 (12.6%) cases, pelvic lymphadenopathy in 124/214 (57.9%), infrarenal para-aortic lymphadenopathy in 34/214 (15.9%), and distant metastases in 12/214 (5.6%).

**Fig. 3:**
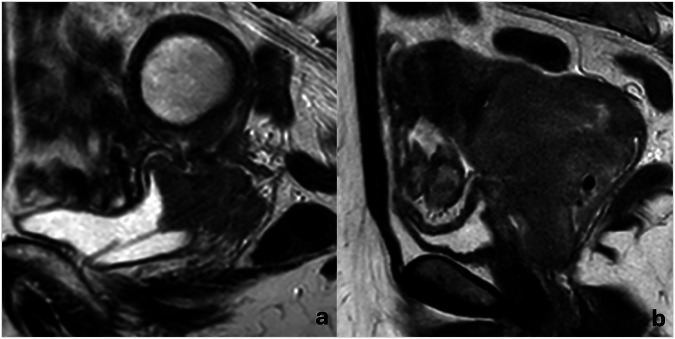
Two cases of MRI-defined bladder wall invasion on sagittal FSE T2-weighted images. **a** A complete loss of neoplasm-bladder cleavage plan, a massive bladder wall thickening with intermediate signal intensity, as well as endoluminal tumor growth, is appreciable; a DJ catheter was placed because of hydronephrosis. **b** An incomplete loss of neoplasm-bladder cleavage plan and a discrete bladder wall thickening with intermediate signal intensity is appreciable, but no endoluminal tumor growth can be observed. The first case was positive at cystoscopy, whereas the second one was negative. Both patients underwent local tumor recurrence (13 and 19 months after chemoradiation, respectively), and both died (25 and 36 months after initial diagnosis, respectively)

Interobserver agreement in CC-bladder wall relationship evaluation showed *k* = 0.890 (95% CI 0.831–0.952) for “loss of the fatty cleavage plane between the neoplasm and the bladder”, *k* = 0.858 (95% CI 0.779–0.942) for “presence of bladder wall thickening”, *k* = 0.851 (95% CI 0.761–0.934) for “loss of the physiological T2-hypointensity of the bladder wall”, and *k* = 0.885 (95% CI 0.786–0.984) for “presence of endoluminal tumor growth”. After consensus, loss of the cervix-bladder cleavage plane was noted in 113/214 (52.8%) cases, bladder wall thickening in 47/214 (22.0%), loss of bladder wall T2-hypointensity in 46/214 (21.5%), and endoluminal tumor growth in 26/214 (12.1%). Bladder wall invasion was considered present in 46/214 (21.5%) patients (Fig. 3) and absent in 175/214 (79.4%). Consequently, 14/214 patients (6.5%) had MRI-stage I neoplasms, 60/214 (28.0%) stage II, 91/214 (42.5%) stage III, and 49/214 (22.9%) stage IV.

In Center A, cystoscopy was performed preoperatively in all 103 patients, while in Center B it was conducted only if bladder wall infiltration was described in the original MRI report or clinically suspected; this occurred in 26/111 (23.4%) cases, while in the remaining cases urine cytology was performed. Endoscopic biopsy was positive for bladder mucosa infiltration in 9/129 (7.0%) patients and negative in 120/129 (93.0%), while urine cytology was negative in all 85/85 (100%) cases, when performed. All patients (9/9, 100%) with bladder mucosa infiltration on cystoscopy had bladder wall invasion on MRI, whereas 35/44 (79.5%) patients with MRI-defined bladder wall invasion had no mucosal infiltration on cystoscopy.

According to the original clinical staging and radiological reports, 163/214 (76.1%) patients were treated with concurrent chemoradiotherapy as their first therapeutic approach, 35/214 (16.4%) underwent surgery, and 16/214 (7.5%) received palliative treatments. Clinically significant treatment-related complications, i.e., complications determining a change in the patient’s oncological management, were observed in 16/214 (7.5%) patients, including 5/214 (2.3%) vesicovaginal fistulas (VVFs).

The median follow-up was 32 months (IQR 13–55). During follow-up, 100/214 (46.7%) patients experienced disease recurrence, with a median time to recurrence of 10 months (IQR 6–22). The primary site of recurrence was in the pelvis in 54/100 (54.0%) cases, and distant in 47/100 (47.0%). During follow-up, 57/214 (26.6%) patients died, with a median time to death of 27 months (IQR 9–50). Of these, 50/57 (87.7%) deaths were attributed to CC, while 7/57 (12.3%) were due to other causes: acute ischemic stroke in 3, myocardial infarction in 2, metastatic lung cancer in 1, and sepsis in 1.

### Prediction of urinary fistula

Two out of the 154 patients without MRI-defined bladder invasion (1.3%) developed VVFs after treatment, compared to 3 out of the 46 patients with MRI-defined bladder invasion (6.5%) (*p* = 0.081, Fisher’s exact test).

### MRI-defined bladder wall invasion predicts recurrence and mortality

The results of the univariate analysis comparing patients with and without tumor recurrence are reported in Table 2. Among the predictors included in the logistic regression analysis, MRI-defined bladder wall invasion (OR 2.24, 95% CI: 1.01–4.95, *p* = 0.047) was the only independent variable for tumor recurrence. All other variables, including bladder mucosa infiltration at cystoscopy, did not reach statistical significance. The model demonstrated moderate discriminative ability, with an AUC of 0.648, and acceptable overall classification accuracy.

**Table 2:** Univariate analysis results comparing patients with and without tumor recurrence

		No recurrence (*n* = 102)	Recurrence (*n *= 98)	*p*-value
Tumors’ maximum diameter (median, mm)		45 (IQR: 38–57)	57 (IQR: 47–68)	**< 0.0001**
MRI-defined bladder wall invasion (*n*)		14 (13.7%)	32 (32.7%)	**0.0015**
Cystoscopy/cytology-defined bladder mucosa infiltration (*n*)		2 (2.0%)	7 (7.1%)	0.078
Tumor stage (median)		3 (IQR: 2–3)	3 (IQR: 3–4)	**0.0001**
Age (years)		55 (IQR: 47–64)	56 (IQR: 50–68)	0.184
Histological type^a^ (*n*)	**0**	91 (89.2%)	85 (86.7%)	0.590
	**1**	11 (10.8%)	13 (13.3%)	
Tumor grade (*n*)	**1**	5 (4.9%)	5 (5.1%)	0.359
	**2**	58 (56.9%)	46 (46.9%)	
	**3**	39 (38.2%)	47 (48.0%)	
Distant metastases (*n*)		0 (0.0%)	12 (12.2%)	**0.0003**
Pelvic lymphadenopathies (*n*)		59 (57.8%)	65 (66.3%)	0.218
Para-aortic lymphadenopathies (*n*)		12 (11.8%)	22 (22.4%)	**0.0449**
Pelvic and/or para-aortic lymphadenopathies (*n*)		60 (58.8%)	65 (66.3%)	0.274
Upfront surgical treatment (*n*)		15 (14.7%)	11 (11.2%)	0.465

Significant *p*-values are in bold

Fourteen patients with MRI-stage I neoplasms were excluded from the analysis

^a^Histological type: 0 = squamocellular, 1 = adenocarcinoma

The results of the univariate analysis comparing survival and cancer-related deaths are reported in Table 3. Among the predictors included in the logistic regression analysis, MRI-defined bladder wall invasion significantly increased the odds of mortality (OR 3.55, 95% CI: 1.44–8.71, *p* = 0.006), along with older age (OR 1.04 per year, 95% CI: 1.00–1.07, *p* = 0.033), and adenocarcinoma histology (OR 4.37, 95% CI: 1.49–12.84, *p* = 0.007). All other variables, including bladder mucosa infiltration at cystoscopy, did not reach statistical significance. The Hosmer-Lemeshow test indicated good calibration (*χ*² = 9.094, df = 8, *p* = 0.334).

**Table 3:** Univariate analysis comparing survivors and cervical cancer-related deaths

		Survivors (*n* = 143)	Cancer-related deaths (*n* = 50)	*p*-value
Tumors’ maximum diameter (median, mm)		50 (IQR: 40–60)	60 (IQR: 49–83)	**< 0.0001**
MRI-defined bladder wall invasion (*n*)		21 (14.7%)	25 (50.0%)	**< 0.0001**
Cystoscopy/cytology-defined bladder mucosa infiltration (*n*)		2 (1.4%)	7 (14.0%)	**0.0003**
Tumor stage (median)		3 (IQR: 2–3)	4 (IQR: 3–4)	**< 0.0001**
Age (years)		55 (IQR: 47–63)	61 (IQR: 50–77)	**0.0029**
Histological type^a^ (*n*)	**0**	131 (91.6%)	39 (78.0%)	**0.011**
	**1**	12 (8.4%)	11 (22.0%)	
Tumor grade	**1**	6 (4.2%)	4 (8.0%)	0.554
	**2**	75 (52.4%)	24 (48.0%)	
	**3**	62 (43.4%)	22 (44.0)	
Distant metastases (*n*)		0 (0.0%)	12 (24.0%)	**< 0.0001**
Pelvic lymphadenopathies (*n*)		87 (60.8%)	36 (72.0%)	0.158
Para-aortic lymphadenopathies (*n*)		20 (14.0%)	14 (28.0%)	**0.026**
Pelvic and/or para-aortic lymphadenopathies (*n*)		88 (61.5%)	36 (72%)	**0.185**
Upfront surgical treatment (*n*)		18 (12.6%)	3 (6.0%)	0.199

Significant *p*-values are in bold

Fourteen patients with MRI-stage I neoplasms and 7 patients who died for other reasons were excluded from this analysis

^a^Histological type: 0 = squamocellular, 1 = adenocarcinoma

### MRI-defined bladder wall invasion reduces overall survival

**Fig. 4:**
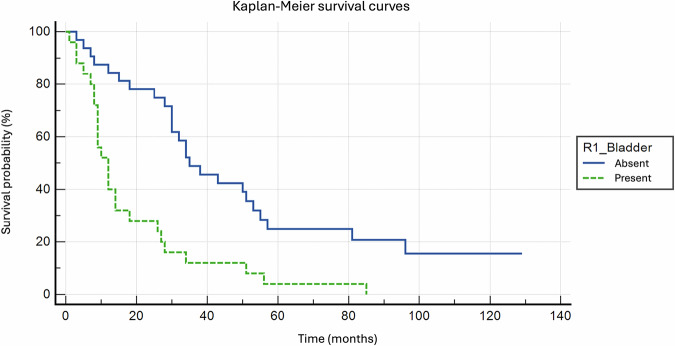
Kaplan–Meier survival analysis among patients with MRI-defined bladder wall invasion (R1_Bladder = Present) and patients without MRI-defined bladder wall invasion (R1_Bladder = Absent). Patients with MRI-defined bladder invasion had a significantly higher risk of mortality compared to those without (HR 3.74, 95% CI: 1.94–7.24, *p* = 0.005)

A survival analysis was conducted to evaluate the impact of MRI-defined bladder wall invasion on cancer-related mortality. The analysis included 200 patients, with 50 cancer-related deaths (24.7%) and 150 censored cases (75.3%), including 7 non-cancer-related deaths. Among the 25 deaths without MRI-defined bladder wall invasion, the mean survival was 51.5 months (SD: 7.3, 95% CI: 37.1–65.8), whereas among the 25 deaths with MRI-defined bladder invasion, the mean survival was 18.8 months (SD: 3.9, 95% CI: 11.1–26.5). The survival curves were compared using the log-rank test, which demonstrated a significant difference in survival between patients with and without MRI-defined bladder wall invasion (*χ*² = 15.40, df = 1, *p* = 0.0001. Patients with MRI-defined bladder invasion had a significantly higher risk of mortality compared to those without (HR 3.74, 95% CI: 1.94–7.24, *p* = 0.005) (Fig. 4).

We did not find a statistically significant difference in survival rates between the 9 patients with both MRI-defined bladder wall invasion and cystoscopy-defined bladder mucosa infiltration and the 37 patients with MRI-defined bladder wall invasion but negative cystoscopy findings using the log-rank test (*χ*² = 2.84, df = 1, *p* = 0.092).

## Discussion

Our study aimed to assess the prognostic impact of MRI-defined bladder wall invasion, i.e., MRI signs of infiltration of any bladder wall layer, detected during preoperative staging of uterine cervical cancer. We included 214 patients with histologically confirmed cervical cancer (CC) who underwent MRI at two gynecologic oncology centers in Italy (Central Hospital, Bolzano, and IRCCS Policlinico Universitario Agostino Gemelli, Rome). Regression analysis revealed that MRI-defined bladder wall invasion is an independent risk factor for tumor recurrence after primary treatment (OR 2.24, 95% CI: 1.01–4.95, *p* = 0.047) and for mortality (OR 3.55, 95% CI: 1.44–8.71, *p* = 0.006). In contrast, cystoscopy-defined bladder mucosa infiltration was not an independent risk factor for either recurrence or mortality. The log-rank test showed that patients with MRI-defined bladder invasion had a significantly higher cancer-related mortality risk compared to those without (HR 3.74, 95% CI: 1.94–7.24, *p* = 0.005).

Interobserver agreement in the assessment of MRI signs of bladder wall invasion was almost perfect for all four evaluated features. However, it should be noted that both readers had substantial experience in pelvic MRI. Multidisciplinary discussions involving highly experienced radiologists are essential to validate these findings among less experienced readers. In our study, signs of bladder wall invasion were relatively common on staging MRI, being present in 21.5% of the patients, while cystoscopy/cytology-defined bladder mucosa infiltration was much rarer, being present in only 7.0% of cases. Notably, none of the patients without MRI-defined bladder wall invasion exhibited bladder mucosa infiltration on cystoscopy/cytology. This finding aligns with previous literature [19, 21, 22, 26] and supports the concept that, to avoid unnecessary additional costs and potential procedure-related morbidity, cystoscopy can be safely omitted in patients without MRI evidence of bladder wall invasion. Conversely, bladder mucosa infiltration was detected by cystoscopy in only 20.5% of the patients showing signs of bladder wall invasion at MRI. This observation is also consistent with prior studies [19, 21, 22, 26], but its clinical significance requires careful interpretation. Indeed, in cervical cancer, bladder mucosa infiltration represents the final stage of a progressive process originating from the outer bladder wall, a region not assessable by cystoscopy. Thus, a negative cystoscopically taken urothelial biopsy doesn’t rule out an even extensive bladder wall infiltration.

Bladder wall invasion is a recognized risk factor for the development of post-radiation VVFs [13, 27, 28]. In our cohort, VVFs occurred rarely, in 2.3% of patients (all treated with concurrent chemoradiotherapy). VVFs were more frequent among patients with MRI-defined bladder wall invasion (6.5%) compared to those without (1.3%), although this difference was not statistically significant (*p* = 0.081, Fisher’s exact test). The limited number of VVF cases likely reduced the robustness of the statistical analysis. Larger studies may be needed to confirm or refute this potential association.

During the follow-up period, which had a median duration of 32 months, 46.7% of patients experienced disease recurrence, a rate within the upper quartile reported in the literature [29, 30], with a median time to recurrence of 10 months. This relatively high recurrence rate may reflect the advanced stage of disease among patients treated in the two centers. Indeed, the median tumor diameter was 52 mm, parametrial invasion was observed in 82.7% of cases, and pelvic lymphadenopathies in 57.9%. Logistic regression analysis identified MRI-defined bladder invasion as a significant independent predictor of recurrence, increasing the odds by 2.2 times, while cystoscopy-defined bladder mucosa infiltration did not emerge as a significant factor. The model demonstrated good calibration and moderate predictive ability, emphasizing the role of bladder invasion and distant metastases in clinical risk stratification.

During the follow-up, 23.4% of patients died due to cervical cancer, with a median time to death of 27 months, consistent with published data [14]. MRI-defined bladder wall invasion emerged as an independent risk factor for cancer-related mortality, increasing the odds of death by 3.6 times. In contrast, cystoscopy-defined bladder mucosa infiltration was not a significant predictor of mortality. This shows that urothelial infiltration assessment does not add meaningful prognostic information when MRI-defined wall infiltration is already present. These findings contradict a study by Nam [28], which reported that the prognosis for patients with cervical cancer and MRI evidence of bladder muscle and/or serosal invasion did not differ significantly from those without such invasion. However, in Nam’s study, the patients’ population (92 patients) was too small to reach statistical significance, and old MRI acquisitions of potentially lower quality were used. Kaplan–Meier survival analysis (Fig. 3) confirmed that MRI-defined bladder wall invasion is associated with significantly reduced survival.

This study has several limitations, primarily related to its retrospective design. Image analysis was performed by two experienced radiologists in consensus, optimizing diagnostic accuracy but necessitating reproducibility testing among less experienced readers. Moreover, not all patients underwent cystoscopy, which is considered the gold standard for assessing bladder mucosa involvement. However, in our series, cystoscopy was negative in all 85 patients who underwent the procedure in the absence of MRI-defined bladder wall invasion. Lastly, the low prevalence of post-treatment VVFs in our cohort limited our ability to thoroughly investigate the impact of MRI-defined bladder invasion on this complication.

In conclusion, our study confirms that MRI can accurately rule out bladder mucosa infiltration in cervical cancer patients, but it also demonstrates that MRI-defined bladder wall invasion has a strong prognostic impact on both disease-free and overall survival, much stronger than that of cystoscopy-defined bladder mucosa infiltration. These findings provide the basis for a possible change in the preoperative staging algorithm of cervical cancer, advocating for MRI as the gold standard for assessing bladder involvement. Indeed, according to our study, cystoscopy can be avoided not only in patients without MRI-signs of bladder wall invasion, as already done in some centers, but also in those showing bladder wall invasion at staging MRI.

## Abbreviations

CC: Uterine cervical cancer
FIGO: International Federation of Gynecology and Obstetrics
VVFs: Vesicovaginal fistulas

## References

[1] Bhatla N, Denny L (2018) FIGO Cancer Report 2018. Int J Gynaecol Obstet 143:2–3. 10.1002/ijgo.12608 30306587 10.1002/ijgo.12608

[2] Zhang Y, Wang C, Zhao Z et al (2023) Survival outcomes of 2018 FIGO stage IIIC versus stages IIIA and IIIB in cervical cancer: A systematic review with meta-analysis. Int J Gynaecol Obstet. 10.1002/ijgo.15218 10.1002/ijgo.15218 37950594

[3] Salvo G, Odetto D, Pareja R et al (2020) Revised 2018 International Federation of Gynecology and Obstetrics (FIGO) Cervical Cancer Staging: a review of gaps and questions that remain. Int J Gynecol Cancer 30:873–878. 10.1136/ijgc-2020-001257 32241876 10.1136/ijgc-2020-001257

[4] Lee SI, Atri M (2019) 2018 FIGO Staging System for Uterine Cervical Cancer: enter cross-sectional imaging. Radiology 292:15–24. 10.1148/radiol.2019190088 31135294 10.1148/radiol.2019190088

[5] Salib MY, Russell JHB, Stewart VR et al (2020) 2018 FIGO Staging Classification for Cervical Cancer: added benefits of imaging. Radiographics 40:1807–1822. 10.1148/rg.2020200013 32946322 10.1148/rg.2020200013

[6] Kido A, Nakamoto Y (2021) Implications of the new FIGO staging and the role of imaging in cervical cancer. Br J Radiol 94:20201342. 10.1259/bjr.20201342 33989030 10.1259/bjr.20201342 PMC9327757

[7] Mansoori B, Khatri G, Rivera-Colón G et al (2020) Multimodality imaging of uterine cervical malignancies. AJR Am J Roentgenol 215:292–304. 10.2214/AJR.19.21941 32551909 10.2214/AJR.19.21941

[8] Fischerova D, Frühauf F, Burgetova A et al (2024) The role of imaging in cervical cancer staging: ESGO/ESTRO/ESP guidelines (update 2023). Cancers (Basel) 16:775. 10.3390/cancers16040775 38398166 10.3390/cancers16040775 PMC10886638

[9] Testa AC, Di Legge A, De Blasis I et al (2014) Imaging techniques for the evaluation of cervical cancer. Best Pract Res Clin Obstet Gynaecol 28:741–768. 10.1016/j.bpobgyn.2014.04.009 24861248 10.1016/j.bpobgyn.2014.04.009

[10] Guimarães YM, Godoy LR, Longatto-Filho A, Reis RD (2022) Management of early-stage cervical cancer: a literature review. Cancers (Basel) 14:575. 10.3390/cancers14030575 35158843 10.3390/cancers14030575 PMC8833411

[11] Gennigens C, De Cuypere M, Hermesse J et al (2021) Optimal treatment in locally advanced cervical cancer. Expert Rev Anticancer Ther 21:657–671. 10.1080/14737140.2021.1879646 33472018 10.1080/14737140.2021.1879646

[12] Quinn MA, Benedet JL, Odicino F et al (2006) Carcinoma of the cervix uteri. FIGO 26th Annual Report on the Results of Treatment in Gynecological Cancer. Int J Gynaecol Obstet 95:S43–S103. 10.1016/S0020-7292(06)60030-1 17161167 10.1016/S0020-7292(06)60030-1

[13] Sun R, Koubaa I, Limkin EJ et al (2018) Locally advanced cervical cancer with bladder invasion: clinical outcomes and predictive factors for vesicovaginal fistulae. Oncotarget 9:9299–9310. 10.18632/oncotarget.24271 29507691 10.18632/oncotarget.24271 PMC5823628

[14] Sammouri J, Venkatesan AM, Lin LL et al (2024) Management and long-term clinical outcomes of patients with stage IVA cervical cancer with bladder involvement. Gynecol Oncol 180:24–34. 10.1016/j.ygyno.2023.11.011 38041900 10.1016/j.ygyno.2023.11.011

[15] Shakur A, Lee JYJ, Freeman S (2023) An update on the role of MRI in treatment stratification of patients with cervical cancer. Cancers (Basel) 15:5105. 10.3390/cancers15205105 37894476 10.3390/cancers15205105 PMC10605640

[16] Pak T, Sadowski EA, Patel-Lippmann K (2023) MR imaging in cervical cancer: initial staging and treatment. Radiol Clin North Am 61:639–649. 10.1016/j.rcl.2023.02.009 37169429 10.1016/j.rcl.2023.02.009

[17] Saleh M, Virarkar M, Javadi S et al (2020) Cervical cancer: 2018 Revised International Federation of Gynecology and Obstetrics Staging System and the Role of Imaging. AJR Am J Roentgenol 214:1182–1195. 10.2214/AJR.19.21819 32182097 10.2214/AJR.19.21819

[18] Ward ZJ, Grover S, Scott AM et al (2020) The role and contribution of treatment and imaging modalities in global cervical cancer management: survival estimates from a simulation-based analysis. Lancet Oncol 21:1089–1098. 10.1016/S1470-2045(20)30316-8 32758463 10.1016/S1470-2045(20)30316-8 PMC7574952

[19] Sapienza LG, Thomas JJ, Showalter TN et al (2022) Endoscopic assessment of radiological stage IVA cervical cancer: a bivariate meta-analysis supporting an evidence-based staging algorithm proposal. Gynecol Oncol 165:642–649. 10.1016/j.ygyno.2022.03.026 35410732 10.1016/j.ygyno.2022.03.026

[20] Agrawal R, Agarwal R (2024) Utility of CT scan in detecting bladder involvement among patients with cervical carcinoma. Cureus 16:e53670. 10.7759/cureus.53670 38455819 10.7759/cureus.53670 PMC10918210

[21] Rockall AG, Ghosh S, Alexander-Sefre F et al (2006) Can MRI rule out bladder and rectal invasion in cervical cancer to help select patients for limited EUA?. Gynecol Oncol 101:244–249. 10.1016/j.ygyno.2005.10.012 16310245 10.1016/j.ygyno.2005.10.012

[22] Knoth J, Pötter R, Jürgenliemk-Schulz IM et al (2020) Clinical and imaging findings in cervical cancer and their impact on FIGO and TNM staging—an analysis from the EMBRACE study. Gynecol Oncol 159:136–141. 10.1016/j.ygyno.2020.07.007 32798000 10.1016/j.ygyno.2020.07.007

[23] Anfinan N (2019) Cervical cancer staging in Saudi Arabia clinicoradiological correlation. Biomed Res Int 2019:8745828. 10.1155/2019/8745828 31341909 10.1155/2019/8745828 PMC6612378

[24] Kakinoki Y, Udo K, Tobu S, Noguchi M (2018) [The role of cystoscopy in the staging of cervical cancer]. Nihon Hinyokika Gakkai Zasshi 109:208–215. 10.5980/jpnjurol.109.208 31631084 10.5980/jpnjurol.109.208

[25] Manganaro L, Lakhman Y, Bharwani N et al (2021) Staging, recurrence and follow-up of uterine cervical cancer using MRI: updated guidelines of the European Society of Urogenital Radiology after revised FIGO staging 2018. Eur Radiol 31:7802–7816. 10.1007/s00330-020-07632-9 33852049 10.1007/s00330-020-07632-9

[26] Hertel H, Köhler C, Elhawary T et al (2002) Laparoscopic staging compared with imaging techniques in the staging of advanced cervical cancer. Gynecol Oncol 87:46–51. 10.1006/gyno.2002.6722 12468341 10.1006/gyno.2002.6722

[27] Khulpateea BR, Paulson A, Carlson M et al (2021) Stage IVA cervical cancer: outcomes of disease related complications and treatment. Int J Gynecol Cancer 31:518–523. 10.1136/ijgc-2019-000386 32920534 10.1136/ijgc-2019-000386

[28] Nam H, Huh SJ, Park W et al (2010) Prognostic significance of MRI-detected bladder muscle and/or serosal invasion in patients with cervical cancer treated with radiotherapy. Br J Radiol 83:868–873. 10.1259/bjr/66646798 20846984 10.1259/bjr/66646798 PMC3473754

[29] Zhao Z, Ruan J, Fang M et al (2024) Efficacy and safety of chemoradiotherapy plus immune checkpoint inhibitors for the treatment of locally advanced cervical cancer: a systematic review and meta-analysis. Front Immunol 15:1459693. 10.3389/fimmu.2024.1459693 39351236 10.3389/fimmu.2024.1459693 PMC11439685

[30] Ka K, Laville A, Rassy E et al (2023) Image-guided adaptive brachytherapy for advanced cervical cancer spreading to the bladder and/or rectum: clinical outcome and prognostic factors. Gynecol Oncol 168:32–38. 10.1016/j.ygyno.2022.11.002 36370612 10.1016/j.ygyno.2022.11.002

